# De novo assembly and characterization of the draft genome of the cashew (*Anacardium occidentale* L.)

**DOI:** 10.1038/s41598-022-22600-7

**Published:** 2022-10-28

**Authors:** Siddanna Savadi, B. M. Muralidhara, Jeffrey Godwin, J. D. Adiga, G. S. Mohana, E. Eradasappa, M. Shamsudheen, Anitha Karun

**Affiliations:** 1 ICAR- Directorate of Cashew Research (DCR), Puttur, D.K., Karnataka 574 202 India; 2 Bionivid Technology Private Limited, 209, 4th Cross Rd, B Channasandra, Kasturi Nagar, Bengaluru, Karnataka 560 043 India

**Keywords:** Biotechnology, Genetics, Molecular biology

## Abstract

Cashew is the second most important tree nut crop in the global market. Cashew is a diploid and heterozygous species closely related to the mango and pistachio. Its improvement by conventional breeding is slow due to the long juvenile phase. Despite the economic importance, very little genomics/transcriptomics information is available for cashew. In this study, the Oxford nanopore reads and Illumina reads were used for de novo assembly of the cashew genome. The hybrid assembly yielded a 356.6 Mb genome corresponding to 85% of the estimated genome size (419 Mb). The BUSCO analysis showed 91.8% of genome completeness. Transcriptome mapping showed 92.75% transcripts aligned with the assembled genome. Gene predictions resulted in the identification of 31,263 genes coding for a total of 35,000 gene isoforms. About 46% (165 Mb) of the cashew genome comprised of repetitive sequences. Phylogenetic analyses of the cashew with nine species showed that it was closely related to *Mangifera indica*. Analysis of cashew genome revealed 3104 putative R-genes. The first draft assembly of the genome, transcriptome and *R* gene information generated in this study would be the foundation for understanding the molecular basis of economic traits and genomics-assisted breeding in cashew.

## Introduction

Cashew (*Anacardium occidentale* L.) is an important perennial nut crop. It belongs to the *Anacardiaceae* family. Currently, it is grown over six million hectares in more than thirty countries. Total raw cashew nut production in the world is more than 3.8 million metric tons with Cote d’Ivoire, India and Vietnam being the major producers^1^. Cashew is believed to be originated in Brazil and its distribution to different parts of the world occurred mainly for soil conservation, afforestation, and wasteland improvement by Portuguese colonials during the sixteenth century^2^.

Cashew is the second most important edible tree nut crop after almonds. Cashew nut kernels are rich in healthy fatty acids and minerals^3,4^. The roasted and salted cashew kernels are consumed as desserts and raw kernels are used in confectionery. The oil extracted from the kernels is being recognized as a good source of vegetable oil and has great use in domestic cooking, cosmetics and pharmaceutics^5–7^. The cashew apple is another edible component of the cashew tree that is processed to prepare products like jam and jellies and its juice is used for preparations of probiotics, ready to serve juices, and alcoholic beverages^8–10^. Cashew nut shell liquid (CNSL), a byproduct of cashew nut processing industry, has great applications in the production of renewable chemicals, materials and energy^11^.

Cashew is a diploid species with a chromosome number of forty-two (2n = 42)^12^. The absolute genome size of cashew determined by flow cytometry is about 0.857 pg/2C (419 Mb/1C) indicating a relatively smaller genome^13^. However, intra-specific variations were observed for absolute nuclear DNA content^13^. Several genetic diversity studies are reported in cashew germplasm from different regions of the world, mainly using dominant markers viz. RAPD, ISSR and AFLP markers^12,14–16^. To date, only a limited number of codominant and sequence-tagged site SSRs markers have been developed in cashew using the microsatellite enriched genomic library screening^17^. Recently, SNP markers were developed in cashew using next generation sequencing (NGS) technologies^18^. A genetic linkage map was constructed in cashew using an F_1_ population of CP 1001 (dwarf clone) × CP 96 (giant clone) with 194 AFLP and 11 SSR markers^19^. However, a comprehensive genetic map with linkage groups equal to the karyotype (n = 21) has not been achieved.

Cashew is susceptible to a number of diseases, such as anthracnose, black mould, angular leaf spot, gummosis and powdery mildew which affect the yields and quality of the produce^20,21^. In this regard, understanding disease resistance mechanisms and the genes involved in durable resistance is needed for developing resistant varieties. Introgression of effective *R* genes in crop cultivars is the most effective and environment friendly means of disease management^22^. However, an understanding of resistance mechanisms and *R* genes is lacking in cashew. Disease resistance genes (*R* genes) play a key role in plant defense through the recognition of pathogen secreted avirulence (Avr) proteins^22^. Cloning and characterization of several *R* genes in different crop plants has revealed that *R* genes share common domains such as nucleotide binding regions (NB), toll-interleukin regions (TIR), leucine rich regions (LRR), coiled coils (CC) and kinases (K). Analyses of whole genome sequences of crop plants provide an opportunity for the identification of candidate *R* genes and thus, provide insights into the R gene evolutions and disease resistance mechanisms, which permits devising novel strategies for breeding resistant crop varieties^23,24^.

In crop plants, genome assembly and genomics research are providing new tools, such as molecular markers and informatics on fundamental mechanisms governing economic traits which are capable of enhancing the efficiency and precision of crop improvement to a great extent^25^. In spite of its economic importance, there are limited genomics resources, so far, no reports of cashew genome assembly, which has hindered molecular biology studies and molecular breeding applications in cashew. In this study, we report the assembly and annotation of the cashew genome for the first time using the hybrid assembly strategy (long reads of Oxford nanopore sequencing and accurate reads from Illumina sequencing). The analysis of assembled genome revealed a set of putative R genes in the cashew genome and also the phylogenetic relationship with the related *Anacardiaceae* species and other plant species.

## Material and methods

### Plant material and nucleic acids (DNA and RNA) extractions

The cashew cultivar Bhaskara, a tall type commercial clone, was used in this study to generate the de novo genome assembly and shoot transcriptome data. Bhaskara is a cashew cultivar developed by ICAR-DCR, Puttur, Karnataka, India (12.45°N latitude, 75.15°E longitude, 90 m above m.s.l.). It is registered under the Protection of Plant Varieties and Farmers' Rights Act (PPV&FR Act) 2001 with registration number 207 of 2019 and is also a reference variety for Distinctness, Uniformity and Stability (DUS) testing in India. The collection and use of plant samples in the present study comply with international, national and/or institutional guidelines.

Leaf tissues and shoots were collected from new flushes and frozen in liquid nitrogen immediately and stored at − 80 °C until use. High quality genomic DNA was extracted from the finely ground leaf tissues using Qiagen Plant Mini kit (QIAGEN, CA, USA). The quality and quantity of the genomic DNA were estimated using Agarose Gel electrophoresis, Nanodrop Spectrophotometer and Qubit fluorometer. Total RNA was extracted from the frozen shoot tissues using Spectrum Plant Total RNA Kit (Sigma, USA). An aliquot of the RNA samples was run on an Agilent RNA Bioanalyzer chip and tape station to check for RNA integrity (Agilent. Technologies, Inc.).

### Library preparation and sequencing

For de-novo hybrid assembly of the cashew genome, genomic DNA libraries suitable for sequencing by Illumina Hi-seq 4000 and Nanopore GridION technology were prepared. Whole genome sequencing (WGS) library was prepared with Illumina-compatible NEXTflex rapid DNA sequencing Bundle (BIOO Scientific, Inc. USA). About 400 ng of Qubit quantified DNA was sheared using Covaris S220 sonicator (Covaris, Inc. USA) to generate a specific fragment range for two Illumina sequencing libraries: (i) 125 to 807 bp (average 466 bp) insert size library and (ii) 264 to 1138 bp (average 700 bp) library. The fragment size distribution was verified on Agilent TapeStation and subsequently purified using HighPrep magnetic beads (MagBio Genomics, Inc, USA). The ends of the purified DNA fragments were repaired, adenylated and ligated to multiplex barcode adaptors following the NEXTFlex Rapid DNA-Seq bundle kit protocol. Illumina Universal adapters used in the study were: 5′-AATGATACGGCGACCACCGAGATCTACACTCTTTCCCTACACGACGCTCTTCCGATCT-3′ and Index Adapter: 5′-GATCGGAAGAGCACACGTCTGAACTCCAGTCAC [INDEX (GCCAAT/CTTGTA)] ATCTCGTATGCCGTCTTCTGCTTG-3′. INDEX–Unique sequence was to identify sample-specific sequencing data. Adapter-ligated DNA was purified using HighPrep beads. Resultant fragments were amplified for five cycles of PCR using Illumina-compatible primers provided in the NEXTflex Rapid DNA sequencing Bundle. The PCR enriched products (sequencing library) were purified with HighPrep beads and quantified by Qubit fluorometer (Thermo Fisher Scientific, MA, USA) and its fragment size distribution was analyzed on Agilent 2200 Tapestation. The Illumina library was paired-end (2 × 150 bp) sequenced on Illumina Hi-seq 4000 following manufacturer guidelines. Oxford Nanopore sequencing library was prepared using a total of 1.3 µg of purified DNA end-repaired (NEBnext ultra II end repair kit, New England Biolabs, MA, USA) and purified using 1X AmPure beads (Beckmann Coulter, USA). Adapter ligation (AMX) was performed at RT (20 °C) for 20 min using NEBnext Quick Ligation Module (New England Biolabs, MA, USA). The adapter ligation reaction mixture was cleaned up using 0.6X AmPure beads (Beckmann Coulter, USA) and the Nanopore sequencing library was eluted in 15 µl of elution buffer supplied with the ligation sequencing kit (SQK-LSK109) from Oxford Nanopore Technology (ONT). Sequencing was performed on GridION X5 (Oxford Nanopore Technologies, Oxford, UK) using SpotON flow cell R9.4 (FLO-MIN106) in a 48 h sequencing protocol on MinKNOW 2.1 v18.05.5.

For transcriptome sequencing, one µg of total RNA was taken for rRNA depletion using the Ribo-Minus Plant rRNA Removal Kit (Plant) and about 20–30 ng of Qubit quantified ribodepleted RNA was used for cDNA synthesis. Purified cDNA was processed further for library preparation as per Illumina-compatible NEBNext Ultra Directional RNA Library Prep Kit (New England Biolabs, Inc.). Sequencing for 150 bp length paired-end (PE) reads was performed in an Illumina HiSeq 4000 (Illumina, USA) to produce 23 million raw sequencing reads.

### De novo genome assembly and annotation

A hybrid assembly approach was employed, in which both Illumina and Oxford Nanopore technology generated reads were used for the genome assembly. The Illumina data were demultiplexing using bcl2fastq and nanopore fast5 data were base-called using Albacore v 2.0.2. The quality of the Illumina data was analyzed using FastQC v.0.11.3^26^ and the adapter sequences were removed using Trimgalore v0.4.0^27^. Quality control of the raw reads was done using fastp v 0.20.0^28^. A quality Phred score cutoff of 30 was used and only high quality reads were retained and used for further downstream analysis.

Genome size was estimated using the K-mer counter, Jellyfish v.2.2.7^29^ and K-mers of size ranging from 51 to 101 were used for the prediction. The results of Jellyfish were processed by GenomeScope2^30^. The raw reads of Illumina and Nanopore sequence data were used for generating hybrid assembly using MaSuRCA v3.3.4, a hybrid genome assembler and a tool that is compatible with Illumina and Nanopore reads, with default parameters^31^. The genome assembly was analyzed for completeness using Benchmarking Universal Single-Copy Orthologs (BUSCO) v.5.4^32^. A highly conserved set of single copy orthologues that were a part of the eudicot_odb10 was used as the database.

For annotation and gene predictions in the assembled genome, a combination of ab initio prediction, homology-based search and transcriptome data was used. The Maker2 pipeline was utilized for gene prediction. It is a wrapper packages that encompass ab initio gene predictors Snap, Augustus, and GeneMark-ES and utilizes transcriptome and protein evidence from related species to predict gene models in the assembly^33^. Transcriptome assemblies generated from cashew tissue (BioSample: SAMN21876806; SRA: SRR16095029) and protein sequences from the species in the *Anacardiaceae* family were downloaded from the NCBI database and used as evidence for the gene prediction. The predicted genes were annotated with NCBI Refseq and Swissprot database proteins using NCBI Blastx + (v2.11)^34^. The predicted transcripts were annotated into Gene Ontology terms, eukaryotic orthologous groups (KOGs) andKEGG pathways^35–38^. The shoot transcriptome reads (SAMN21876806; SRA: SRR16095029) generated by us were used to validate the draft genome assembly. The transcript reads were filtered using fastp and aligned against the draft genome using Hisat2^39^. Further, UCSC pairwise sequence aligner BLAT (DNA database vs RNA query) was used to map the transcripts from the assembled transcriptome to the draft genome^40^.

### Repetitive sequence identification

RepeatModeler version 2.0.1 (http://www.repeatmasker.org/Re peatModeler/) was used to create a de novo consensus library of repeat families using the assembled genome. The repeat library generated by RepeatModeler was used as a custom library for RepeatMasker version 4.0.9_p2 (default parameters) to predict the repeat sequences in the genome^41^.

### Non-coding RNA analysis

Non-coding RNA such as snoRNA, tRNA, rRNA and microRNA were identified using Infernal (v1.1.4)^42^. Infernal uses a homolog based search for RNA structures against the Rfam^43^ database.

### Identification of disease resistance (*R*) genes

The prediction of potential disease resistance (*R*) genes in the de novo assembled cashew genome was performed using the Plant Resistance Genes database (PRGdb 3.0; http://prgdb.org) comprising of curated reference *R* genes with the Disease Resistance Analysis and Gene Orthology (DRAGO v.2) pipeline^44^. DRAGO v.2 uses COILS 2.2 and TMHMM 2.0c at the backend to detect Coiled Coil regions and transmembrane domains, respectively, which are used to identify and classify the genes into different classes of R-genes.The R-genes of Mango, Pistachio and Arabidopsis were also obtained using Disease Resistance Analysis and Gene Orthology (DRAGO 2) pipeline.

### Anchoring of microsatellite markers on the draft genome

Twenty one polymorphic microsatellite loci are reported by Croxford et al. (2006) in cashew. The primer sequences of these microsatellite markers were anchored on the draft genome using the PatMaN aligner^45^. PatMaN is uniquely developed for mapping short nucleotide sequences onto large sequence databases. The number of mismatches allowed was 2 and no gaps were allowed. An alignment was considered to be genuine if both the forward and reverse primers mapped to the same location in the genome within a maximum range of 500 bp.

### Phylogenetic analyses, Expansion and Contraction of gene families

OrthoFinder^46^ was used to identify orthologous groups in rice, *Arachis* sp, mango, pistachio, apple, Arabidopsis, *Prunus* sp, *Populus* sp and cashew draft genome. The phylogenetic relationship of *A. occidentale* between the four other species was determined by doing a multiple sequence alignment of the proteins with the MUSCLE aligner^47^. Further, the divergence of the species was estimated by the Reltime Maximum likelihood (Jones-Taylor-Thornton substitution model) method described by Tamura et al.^48^ using the MEGA X software. The single copy orthologous (orthogroups that contain one gene for each species) was used to generate a STAG^49^ phylogeny tree and rooted using STRIDE^50^. An analysis of gain and loss of gene families based on the orthology was undertaken using Computational Analysis of gene Family Evolution v5 (CAFE5)^51^. The orthologous groups which were determined to be expanding or contracting (p-value significance at 0.05) were taken forward and the KEGG pathways enriched in these groups were determined using the KOBAS web server^52^.

## Results and discussion

### Genome sequencing and assembly

A total of 47 million of 264–1138 bp (average 700 bp) insert (representing 34 × genome coverage) and 95 million of 125–807 bp (average 466 bp) insert (representing 68.7 × genome coverage) Illumina reads were generated providing a total coverage of 102.7 × coverage (of expected 419 Mb). Subsequently, 3.6 million reads of Oxford Nanopore sequence providing a 19 × coverage was generated from whole genome libraries with an average read length of 2.21 kb, median read length of 1.35 kb and an N50 value of 3.68 kb and a maximum read length of 81.88 kb. A de novo assembly of cashew genome by hybrid assembly strategy using both short Illumina reads and long Oxford Nanopore reads generated a 356.6 Mb genome corresponding to 85% of the average genome size of 419 Mb (ranging from 360 to 440 Mb among different cashew accessions) estimated by flow cytometry^13^. Further, k-mer analysis using the Illumina read sequences showed that the estimated genome size ranged from 330 to 345 Mb, which is similar to the size of the assembled draft genome. The total number of scaffolds generated in the hybrid assembly was 3268. The N50 value of the genome assembly was 420 kb and the maximum scaffold length was 1.81 Mb (Table 1). Further, the NG50 value of assembled genome is 340 kb considering 419 Mb as the estimated genome size. The GC and AT content of the genome sequence reads was 33.64% and 65.91%, respectively. The assembled genome of the cashew tree is submitted to the NCBI database (BioProject ID: PRJNA766521). A relatively large number of scaffolds and lower N50 could be due to the fact that our genome assembly is mostly based on short read Illumina sequences data, with limited long reads data from Oxford Nanopore sequencing^53^.

**Table 1:** Assembly statistics of the *A. occidentale* genome.

Hybrid assembly	Statistics
Assembly size (bases)	356,594,228 (356 Mb)
Number of scaffolds	3268
N50 value	420,659
Longest scaffold (bases)	1,810,141
Shortest scaffold (bases)	1001
Average scaffolds length (bases)	109,117
Median scaffolds length (bases)	20,599.5
(A + T)s	65.91%
(G + C)s	33.64%
Ns	0.45%
Scaffolds longer than 1 Kb	3268
Scaffolds longer than 10 Kb	2275
Scaffolds longer than 1 Mb	42
BUSCO evaluation (% completeness)	91.8%

Assessing the BUSCO sets in the de novo assembled genomes allows the quantitative assessment of genome completeness based on the evolutionarily informed expectations of gene contents from the near-universal single-copy orthologs^54^. Evaluation of the de novo assembled cashew genome for completeness was performed with the BUSCO pipeline using the eudicot database (eudicot_odb10). BUSCO analysis identified 2135 complete BUSCOs (91.8%) out of 2326 BUSCO groups of the eudicot_odb10 database (Table 2). Among the complete BUSCOs, 77.9% were single-copy orthologs, 12.7% were duplicated orthologs, and 0.9% were fragmented. The number of missing BUSCOs in the assembled cashew genome was 170 (7.2%).

**Table 2:** Summary of the BUSCO assessment for *A. occidentale* genome.

Eukaryota_odb10	Num	%
Complete BUSCOs (C)	2135	91.8
a. Complete and single-copy BUSCOs (S)	1835	78.9
b. Complete and duplicated BUSCOs (D)	300	12.9
Fragmented BUSCOs (F)	21	0.9
Missing BUSCOs (M)	170	7.3
Total BUSCO groups searched	2326	

Anchoring sequence tagged markers on the draft genome can be another level of validation for the draft genome. PatMaN is uniquely developed for mapping short nucleotide sequences onto large sequence databases. PatMan analysis showed that 18 of the 21 markers were mapped to the genome without any mismatches and one locus (mAoR11c) was mapped with a single mismatch on the reverse primer (Supplementary Table S1).

### Gene annotation and Functional classification

A total of 31,263 genes coding for a total of 35,000 gene isoforms (transcripts) were predicted (Table 3; Supplementary Table S1). The number of protein-coding genes in the assembled cashew genome is similar to that found in *Pistacia vera*^55^ but slightly higher than that reported in mango, a member of the *Anacardiaceae* family^56^. The average gene size was 3185 nucleotides (nt), with an average of 5.75 exons per gene. The average exon size was 258 nt and the average intron size was 371 nt (Table 3). The GC content of exonic regions was 42.01%, which is higher than that in the intronic regions of genes (31.98%; Table 3).

**Table 3:** Annotation statistics for *A. occidentale* genome.

Number of putative protein-coding genes	31,263
Number of putative gene isoforms/transcripts	35,000
Total gene length	99,592,494 bp (99.5 Mb)
Average gene size (nt)	3185
Average number of exons/gene	5.75
Total exon length	46,432,819 bp (46.5 Mb)
Average exon length (nt)	258
GC content of exons (%)	42.01
Average number of introns/gene	4.7
Total intron length	54,613,478 bp (54.6 Mb)
Average intron length (nt)	371
GC content of introns (%)	31.98

**Figure 1:**
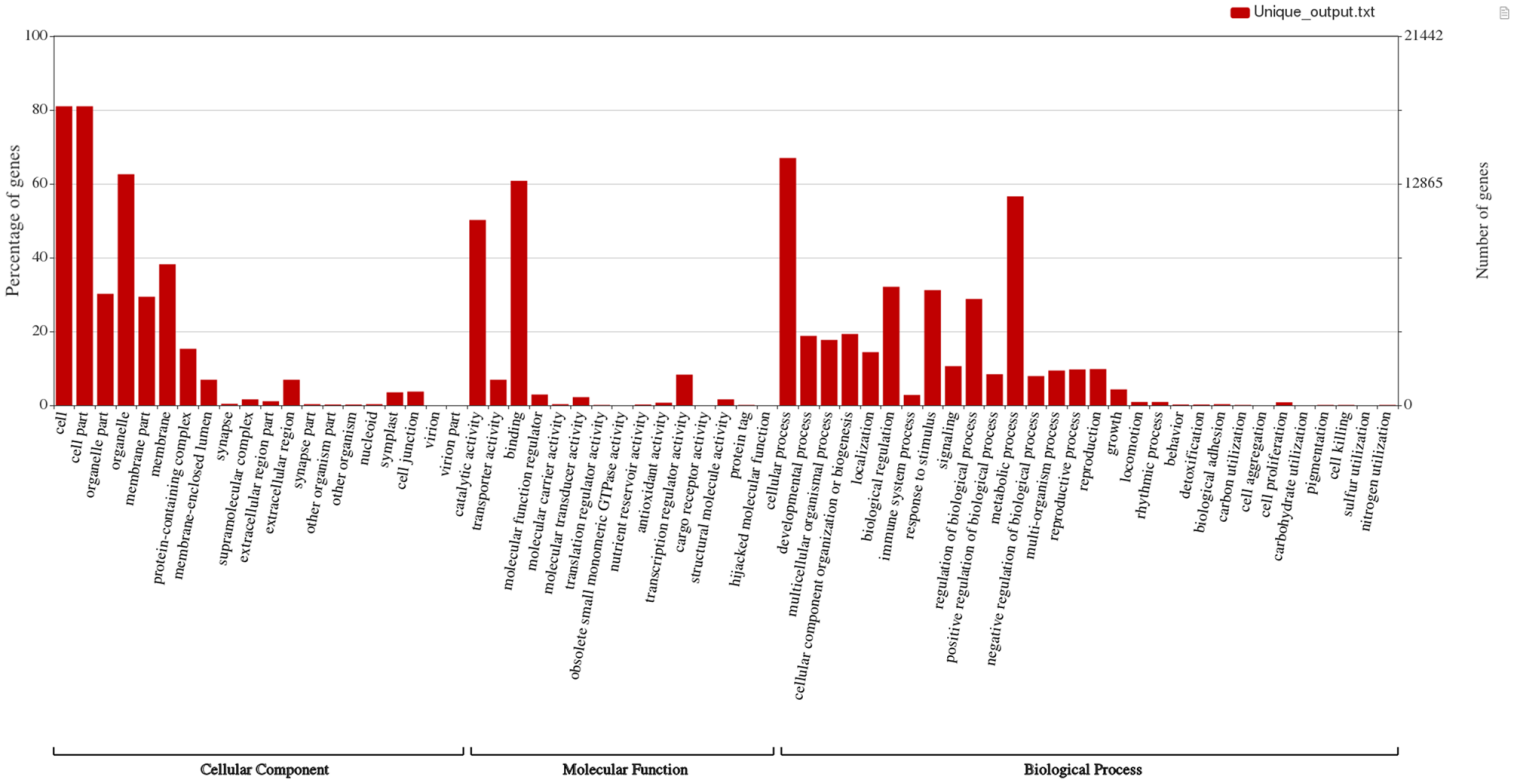
GO ontology annotation of cashew genome showing the major GO terms in each of the three categories of Molecular Function, Biological Process and Cellular Component. The left-hand Y-axis indicates the percentage of genes in a specific sub-category of each main category. The right-hand Y-axis represents the number of genes in a sub-category. Proportions were calculated using only the values of the major GO terms in each category.

The predicted gene sequences were annotated with Gene Ontology (GO) terms for functional classification. The predicted genes in the cashew genome were assigned to 57,004 GO terms, as in many cases, one gene sequence was assigned to multiple GO terms (Supplementary Table S1). A total of 5673 unique GO terms were found and were classified into three main categories: cellular components, biological processes and molecular functions. In the predicted genes, 28,350, 23,450 and 19,600 genes were assigned to at least one GO term in the cellular components, biological pathways and molecular function categories, respectively. The genes were further classified into 57 functional groups in the three main categories (Fig. 1, Supplementary Table S1, S3).

**Figure 2:**
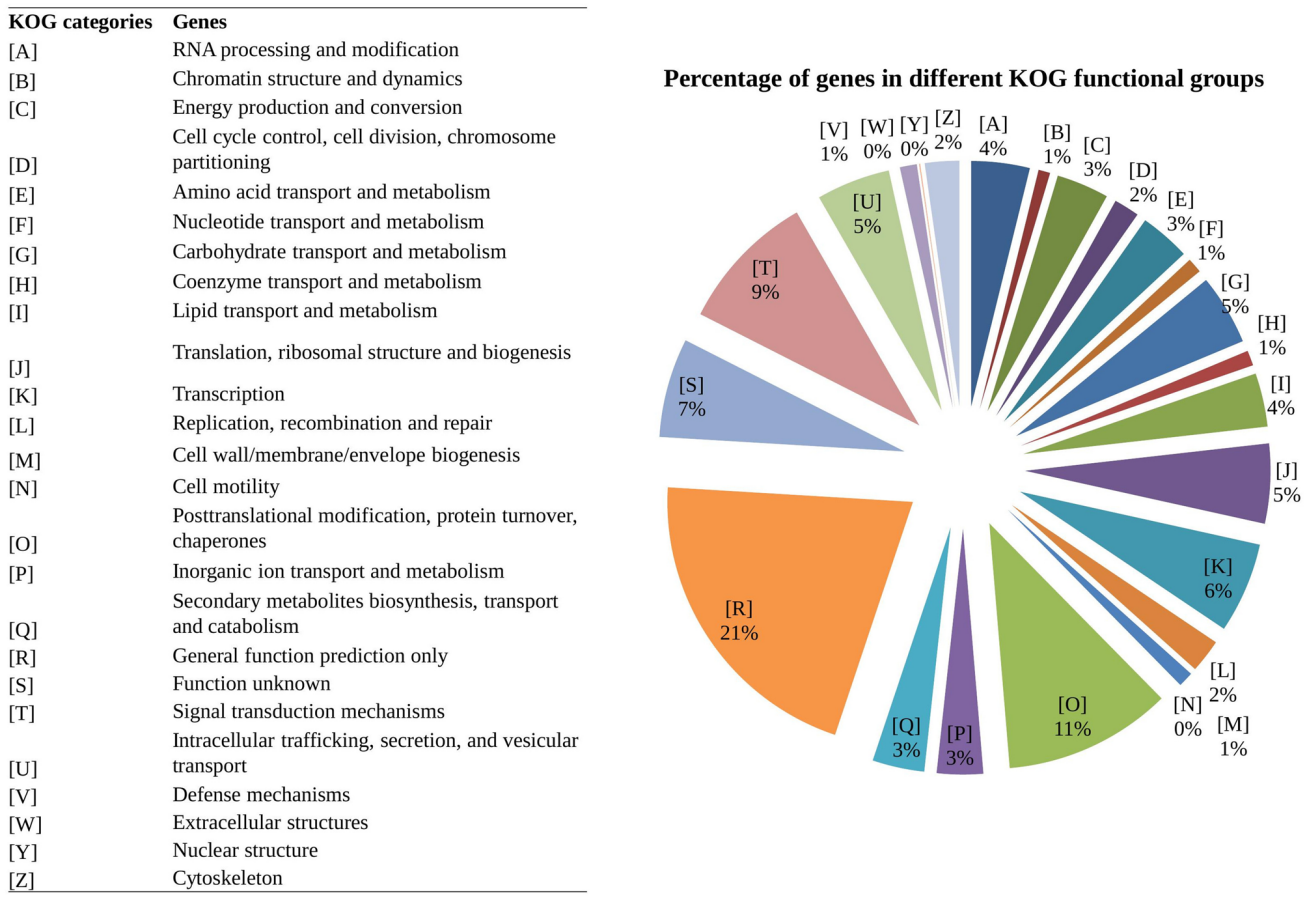
EuKaryotic Orthologous Groups (KOG) functional classification of cashew genome showing the percentage of genes in the 24 functional classes of KOG database.

All the predicted genes were also annotated and classified according to EuKaryotic Orthologous Groups (KOG) categories. A total of 15,287 genes were annotated and grouped into 25 functional categories of KOG (Fig. 2, Supplementary Table S2). Among the 25 functional categories, “General function prediction only” (20.80%) and “Posttranslational modification, protein turnover, chaperones” (11.04%) followed by “Signal transduction mechanisms” (9.21%) categories dominated in the KOG annotation and classified. About 6.53% of the KOG annotated genes were categorized as “function unknown” (Supplementary Table S2).

**Figure 3:**
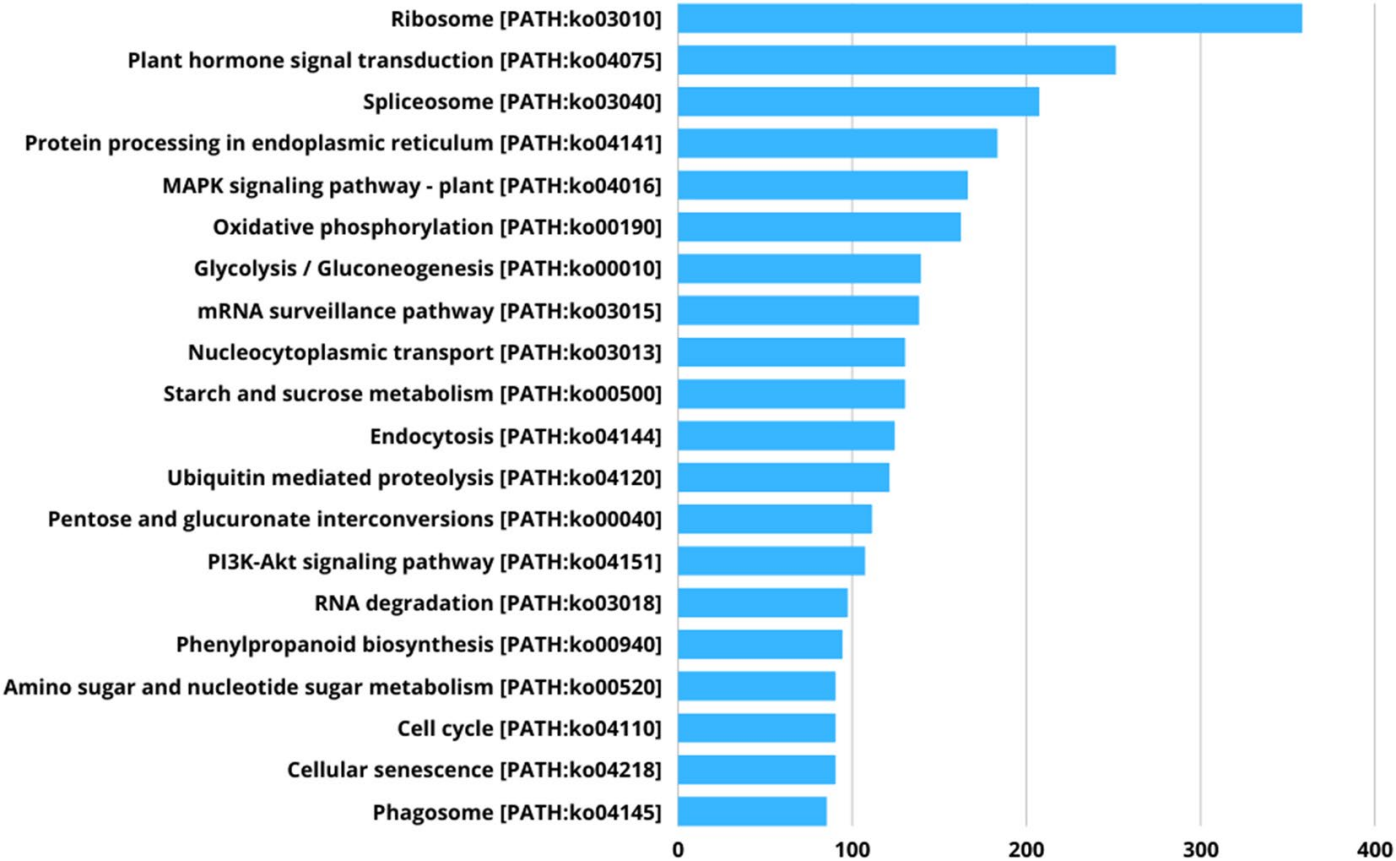
KEGG analysis of cashew genome showing the top 25 highly represented KEGG pathways. The X-axis indicates the KEGG pathways and the Y-axis indicates the number of transcripts in each pathway.

The metabolic pathways annotations were carried out based on the KEGG database using the KEGG Automatic Annotation Server (KAAS)^37^. The KAAS analysis first provided every submitted sequence with KEGG orthology (KO) identifiers and then, the metabolic pathways were identified based on the KO number^36,37^. The KEGG pathway analysis revealed diverse pathways with “Ribosome,” “Phytohormone signal transduction,” “Spliceosome,” “Protein processing in ER” and “Mitogen-activated protein kinase (MAPK) signaling pathway” as the five most highly represented pathways (Fig. 3, Supplementary Table S1).

In addition to coding sequences, 1361 non-coding RNAs (ncRNAs) were identified in the genome assembly using the Infernal (v1.1.4). Of it, tRNAs (567), snoRNAs (400) and micro RNAs (225) were predominantly present (Supplementary Table S4). ncRNAs play a role in gene regulation at the transcriptional and post-transcriptional levels^57^. Some ncRNAs are involved in the epigenetic regulation of gene functions, and miRNAs are one such type of ncRNAs. miRNAs are known to play important roles in plant development and stress responses^58^. Hence, in recent times, focused efforts are being made for miRNA identification. However, the traditional miRNA identification methods are reliant on RNA sequencing, miRNAs may not be captured as it is often biased towards abundant transcripts and when the miRNAs are produced in a tissue and developmental stage specific manner^59,60^. Therefore, ab initio /de novo miRNA identifications using genome sequences will complement the miRNAs identification processes in plant genomes^61,62^.

Transcriptome sequences of shoots (SAMN21876806; SRA: SRR16095029) aligned against the draft genome showed an alignment of 97% (22618536 reads out of 23560975). Further, 73% (52120) of the transcripts were mapped to the genome with an identity of 70% and higher.

### Repeat sequence identification

Analysis of the assembled genome of the cashew tree showed that 46.34% (165 Mb) of the genome comprised of repetitive sequences (Table 4). The analysis of composition of sequence repeats showed that interspersed or transposable elements were dominant (44.94% of the assembly), of which long terminal repeat (LTR) retrotransposons were the major component (19.41% of the assembly) (Table 4). The proportion of repetitive elements in the cashew genome is smaller compared to the 70% in *Pistacia vera*, a member of *Anacardiaceae *family, draft genome suggesting that repetitive elements increase with an increase in genome sizes^55^. However, greater proportions of long terminal repeats (LTRs) and unclassified elements which are similar to that are found in the *P. vera*^55^ and other plants^63^. In general, the mobility of transposons and retroelements contributes to the expansion and evolution of plant genomes and may alter gene expressions by epigenetic modifications^64,65^. Thus, understanding the repeat sequences in genomes can facilitate the characterization of their role in cashew genome evolution and phenotypes.

**Table 4:** Summary of repeat elements identified in *A. occidentale* genome.

Elements	Number	Length (bases)	% of the assembly	% of total repeats
DNA elements	15,790	8,457,738	2.37	5.11
LINEs	4742	3,113,630	0.87	1.88
LTRs	61,102	69,215,937	19.41	41.89
others	187,296	79,472,321	22.29	48.10
Total interspersed		160,259,626	44.94	96.97
Simple sequence repeats	95,867	3,807,279	1.07	2.31
Small RNA	213	208,766	0.06	0.13
Low complexity	19,048	964,998	0.27	0.58
Total Repeats			46.34	100

### Candidate disease resistance (***R***) genes

Plant defenses against most pathogens are initiated by disease resistance (*R*) genes. Plant genomes comprise of several *R* genes that encode different classes of proteins that provide resistance against pathogens^66^. Analysis of 35,000 predicted protein sequences for resistance (*R*) genes related domains and motifs showed that 3104 proteins comprised *R* gene related domains (Supplementary Table S5). Of these, 647 R proteins contained a single domain, 1692 R proteins contained double motifs, 641 R proteins contained three domain types, 122 R proteins contained four domain types and two R proteins contained five types of domains. Majority of the R proteins comprised TM-kinase domains (782) followed by NBS-TM (336), Kinase (269) and other domain types (Table 5). Among the different *R* gene classes, Kinases (KIN) (33%), NBS (N) (14.5%) and Receptor Like Proteins (RLP) (13%) were dominant (Supplementary Table S5). These results are similar to the *R* genes predicted in other plants genomes^67,68^.

**Table 5:** Prediction of *R* genes domains/motifs present in the proteins identified from the *A. occidentale* genome using DRAGO v.2 and Plant resistance gene database.

Domain/motif types	Number of proteins	Domain/motif types	Number of proteins
TM-Kinase	782	CC-NBS-TM-LRR	17
NBS-TM	336	NBS-CC	17
Kinase	269	CC	15
TM-LRR	197	NBS-CC-TM-LRR	15
LRR-TM	194	CC-NBS-LRR-TM	13
LRR	166	CC-LRR-TM-Kinase	12
CC-TM-Kinase	136	NBS-CC-LRR-TM	11
TM-Kinase-LRR	130	CC-TIR	9
LRR-Kinase-TM	93	CC-NBS-LRR	5
TM	88	LRR-Kinase	5
NBS	78	NBS-Kinase	5
CC-NBS-TM	52	Kinase-TM	4
NBS-CC-TM	50	LRR-TM-TIR	3
CC-TM-LRR	44	NBS-CC-LRR	3
LRR-TM-Kinase	40	NBS-LRR	3
CC-Kinase	36	NBS-TM-TIR	3
TM-TIR	35	CC-LRR-Kinase	1
TIR	32	CC-NBS-LRR-TM-Kinase	1
CC-LRR	30	CC-NBS-TM-Kinase	1
CC-TM-Kinase-LRR	26	NBS -TM	1
CC-LRR-Kinase-TM	25	NBS-CC-LRR-TM-TIR	1
CC-LRR-TM	23	NBS-CC-TM-Kinase	1
NBS-TM-LRR	21	NBS-LRR-Kinase-TM	1
CC-NBS	19	NBS-TIR	1
CC-TM	19	NBS-TM-Kinase-LRR	1
NBS-LRR-TM	19	TM-TIR-LRR	1
NBS-TM-Kinase	19	-	-
Total number of proteins			3104

The NBS-LRR domain is one of the most characterized domains in plant resistance against pathogens. Hence, genes containing the NBS-LRR, CC-NBS-LRR and TIR-NBS-LRR domains of Mango, Pistachio and Arabidopsis derived from DRAGO2 pipeline were compared with cashew (Supplementary Table 5). Cashew showed a lower number of *R* genes containing these domains compared to other organisms.

### Phylogenetic analysis, Expansion and contraction of gene families

**Figure 4:**
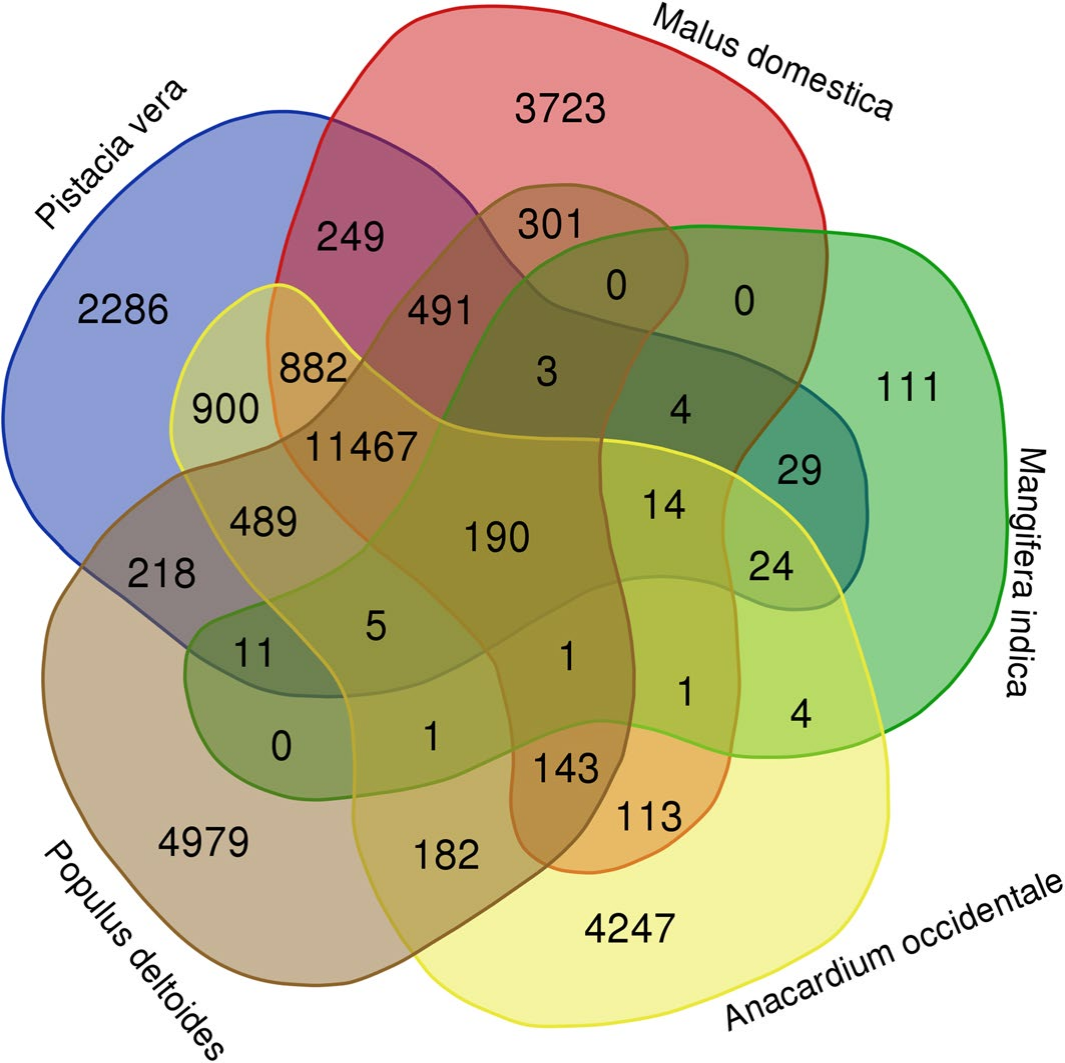
Venn diagrams displaying clusters of shared and unique orthologous gene families in the *A. occidentale* and four tree species (*M. indica*, *P. vera*, *P. deltoides*, *M. domestica*).

**Figure 5:**
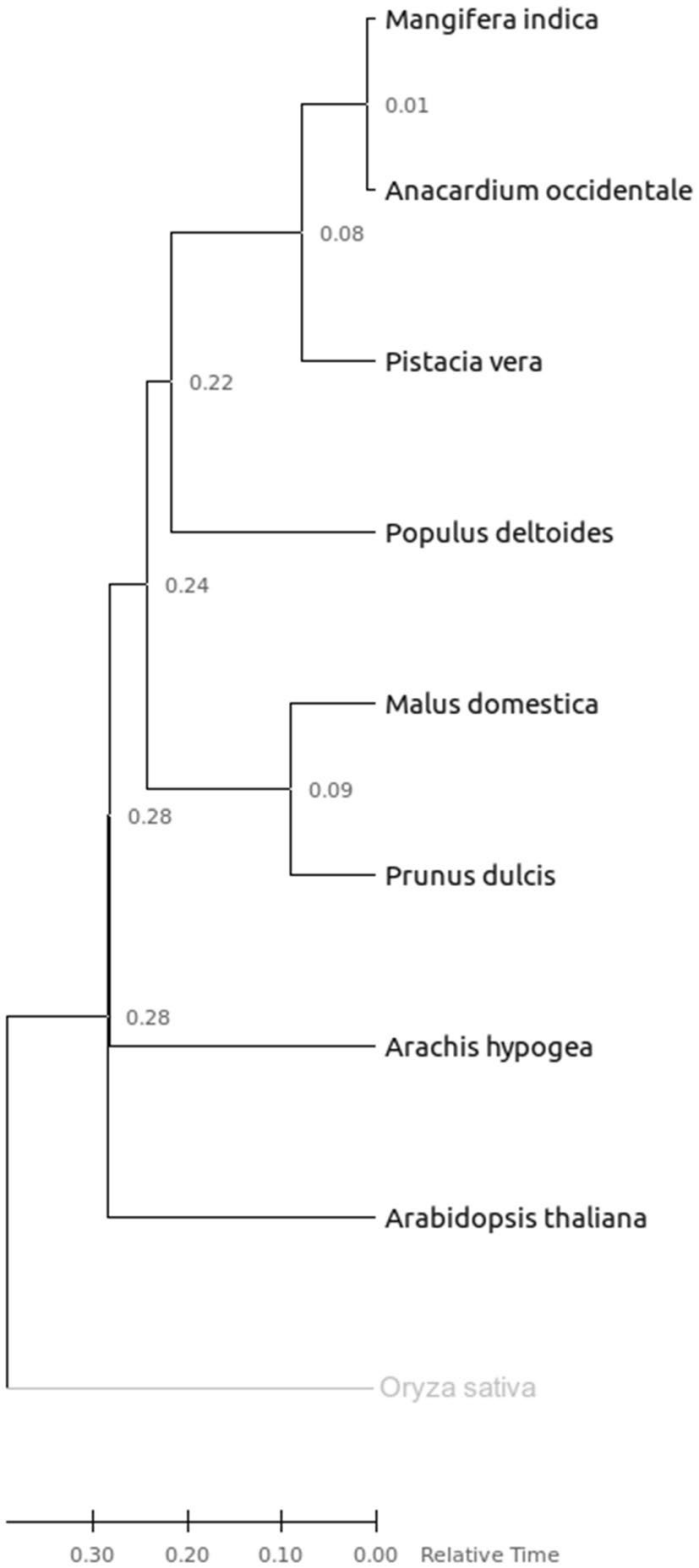
Phylogenetic tree of *A. occidentale* and nine plant species based on protein sequences of single-copy orthologous genes. The numbers at the nodes of the phylogenetic tree show the relative divergence times between species calculated using the RelTime method. The scale reflects the divergence scale in terms of the number of substitutions per site (amino acids).

The evolutionary relationship among *A. occidentale, A. thaliana, A. hypogea, O. sativa, M. domestica, M. indica, P. vera, P. deltoides* and *P. dulcis* were analyzed using multiple sequence alignment of the proteins with the MUSCLE aligner. OrthoFinder uses an all-vs-all Diamond blast search of the proteins from the species to identify orthologous protein groups between species. OrthoFinder analysis showed that of 398,813 proteins from five species, 376,630 (94.44%) were clustered into 31,224 orthologous groups (Supplementary Table S6). Species specific groups and common orthologous groups in the five tree species tested are represented in the Venn diagram (Fig. 4). Pairwise genetic distance analysis showed that genetic distance between the *A. occidentale* and *M. indica* was the lowest (0.12) followed by *P. vera* (0.13), *P. dulcis* (0.38), *P. deltoids* (0.38), *M. domestica* (0.39), *A. hypogea* (0.39), *A. thaliana* (0.45) and *O. sativa* (0.59). In the phylogenetic tree, *O. sativa*, a monocot formed an outgroup from the dicot species. In the dicot species, the *Anacardiaceae* members *M. indica*, *P. vera* and *A. occidentale* formed a clade that is separated from clades of other dicot species (Fig. 5). Among the *Anacardiaceae* members, *M. indica* and *A. occidentale* were found to be closer compared to the *P. vera*, which is in corroboration with the results of the studies on generic relationships in *Anacardiaceae* species based on the 81 morphology, anatomy, palynology and chemotaxonomy related characters and housekeeping genes sequence data analysis^69^. In phylogenetic relationships, the estimation of divergence times is helpful in understanding the evolutionary lineage. The estimation of divergence times based on molecular data is considered an intricate activity. However, the RelTime method permits the estimation of biological timescales i.e., divergence times in a straightforward and faster way, even using large genome data sets. In this study, the relative divergence times among the nine species were calculated using the RelTime method and are shown in the Fig. 5. The phylogeny provides an account of life in the past and also acts as a powerful predictive tool for basic and applied research in crop plants. Analysis of plant genomes provides signatures of evolutionary history in plants. Phylogenetic studies based on genomic studies allow proper taxonomic classification of species with ambiguity, and act as a guide for crop improvement and conservation^70^.

**Figure 6:**
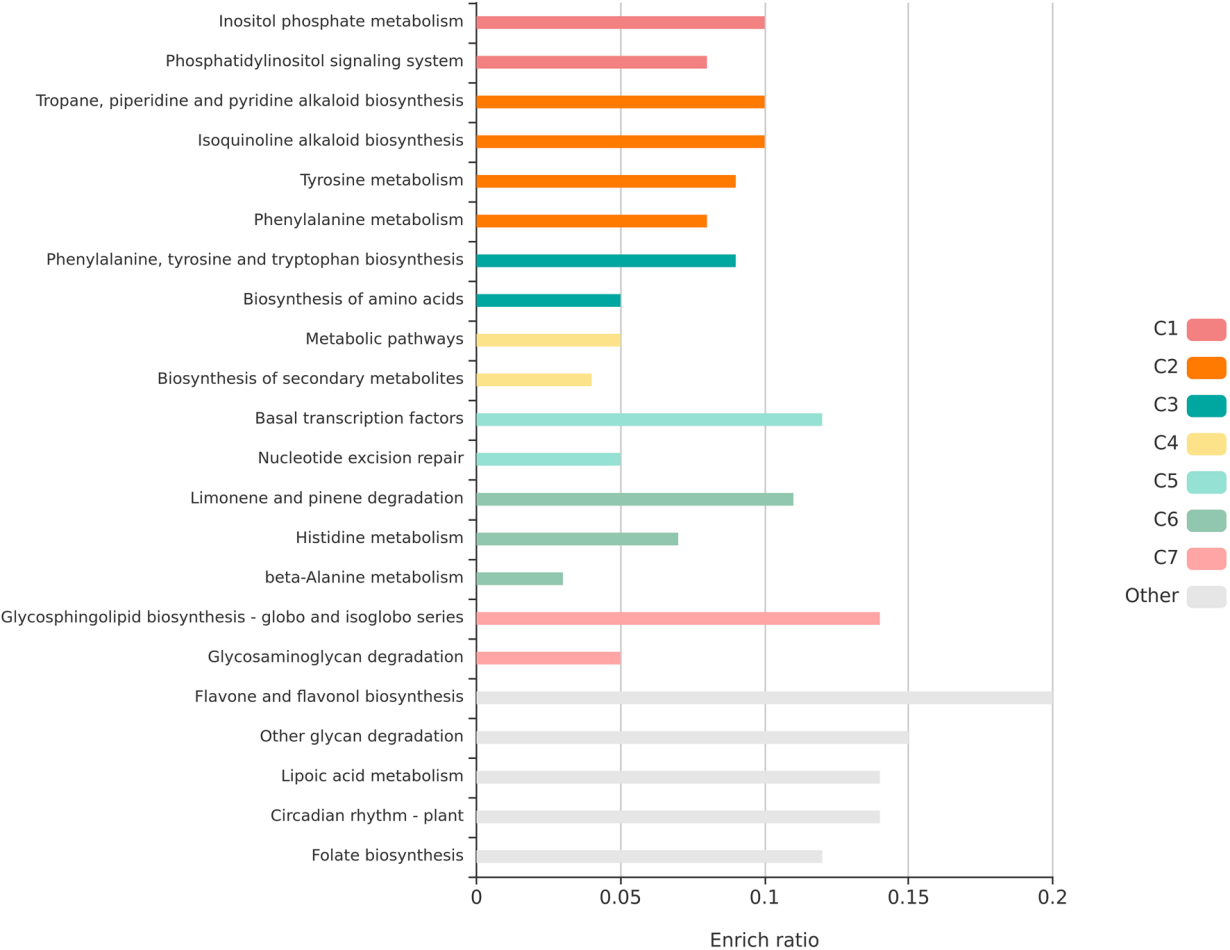
Statistically enriched pathways in the 1586 orthologous groups which were expanding or contracting among the species identified using the KOBAS database. The Y-axis indicates the pathway name and the X-axis indicated Enrich ratio. Enrich ratio is the proportion of the number of differentially expressed genes or proteins in the pathway to the total number of annotated genes or proteins in the pathway. The greater the Enrich ratio, the greater will be the degree of pathway enrichment.

In the analysis of expansion and contraction of gene families, according to the gamma model in CAFE5, 1586 of the groups had a statistically significant expansion or contraction. In cashew, an expansion of 839 and a contraction of 23 groups was observed. This provides an inference that the gene families are evolving differently among the compared organisms^71^. The KEGG pathways enriched in the 1586 orthologous groups which were expanding or contracting (p-value significance at 0.05) showed that flavones and flavonol biosynthesis and other glycan degradation pathways were the two largest enriched groups (Fig. 6, Supplementary Table 7). The pathway enrichment analysis provides a mechanistic view of the gene lists derived from genome-wide data and it permits more insights into the biological processes^52^.

## Conclusions

In this study, we report for the first time a de novo assembled draft genome of *A. occidentale* cultivar Bhaskara by a hybrid assembly of Illumina reads and Oxford Nanopore reads data. The draft assembly generated is 356 Mb in size with a scaffold N50 length of 420 kb. The completeness of assembly analyzed using BUSCO analysis showed 91% of genome completeness. We predicted a total of 31,263 genes coding for a total of 35,000 gene isoforms (transcripts) from the assembled cashew genome. Analysis of the identified protein sequences for candidate resistance (*R*) genes showed the presence of 3104 candidate *R* genes and Kinases as the dominant class of *R* genes in the cashew genome. The phylogenetic analyses using the single-copy orthologous genes revealed that *A. occidentale* is very close to the *M. indica*, which is an *Anacardiaceae* family tropical tree among the nine species compared. The genome assembly, annotation and mining of candidate *R* genes from the cashew genome in this study will be useful for the development of a large set of genome-wide markers, conducting molecular studies and marker-assisted breeding programs for the improvement of yield, quality and tolerances to stresses in cashew.

## Supplementary Information

Supplementary Table S1.

Supplementary Table S2.

Supplementary Table S3.

Supplementary Table S4.

Supplementary Table S5.

Supplementary Table S6.

Supplementary Table S7.

Supplementary Figure S1.

Supplementary Figure S1.

Supplementary Figure S1.

Supplementary Figure S1.

Supplementary Figure S1.

Supplementary Figure S1.

## Data Availability

Sample information and sequencing data have been uploaded on Genbank (BioProject: PRJNA766521 contains Whole genome data and BioProject: PRJNA766798 contains transcriptome data). Oxford Nanopore sequencing reads have been deposited at (BioSample: SAMN21850282; SRA: SRR16094803) and Illumina short reads at (BioSample: SAMN21850283, SAMN21850284; SRA: SRR16094803, SRR16094803). Transcriptome sequencing data is available at (BioSample: SAMN21876806; SRA: SRR16095029).

## References

[1] INC (2021). International Nut and Dried Fruit Council Statistical year book-2021. p. 21.

[2] Bhoodes, R. K., & Francis, C. A. The Transition of World Cashew Industry and the Challenges to India (Doctoral dissertation, Cochin University of Science and Technology) (2014).

[3] Rico R, Bulló M, Salas-Salvadó J (2016). Nutritional composition of raw fresh cashew (*Anacardium occidentale* L.) kernels from different origin. Food Sci. Nutr..

[4] Bai SH, Brooks P, Gama R, Nevenimo T, Hannet G, Hannet D, Hannet D, Randall B, Walton D, Grant E, Wallace HM (2019). Nutritional quality of almond, canarium, cashew and pistachio and their oil photooxidative stability. J. Food Sci. Technol..

[5] Athar M, Nasir SM (2005). Taxonomic perspective of plant species yielding vegetable oils used in cosmetics and skin care products. Afr. J. Biotechnol..

[6] Yahaya AT, Taiwo O, Shittu TR, Yahaya LE, Jayeola CO (2012). Investment in cashew kernel oil production; cost and return analysis of three processing methods. Am. J. Econ..

[7] Emelike NJT, Akusu MO, Ujong AE (2017). Antioxidant and physicochemical properties of oils extracted from cashew (*Anacardium occidentale* L.) kernels. Int. J. Food Sci..

[8] Marques de Carvalho J, Maia GA, Wilane de Figueiredo R, Sousa de Brito E, Rordrigues S (2007). Development of a blended beverage consisting of coconut water and cashew apple juice containing caffeine. Int. J. Food Sci..

[9] Pereira ALF, Maciel TC, Rodrigues S (2011). Probiotic beverage from cashew apple juice fermented with Lactobacillus casei. Food Res. Int..

[10] Gamero A, Ren X, Lamboni Y, de Jong C, Smid EJ, Linnemann AR (2019). Development of a low-alcoholic fermented beverage employing cashew apple juice and non-conventional yeasts. Ferment.

[11] Mgaya J, Shombe GB, Masikane SC, Mlowe S, Mubofu EB, Revaprasadu N (2019). Cashew nut shell: a potential bio-resource for the production of bio-sourced chemicals, materials and fuels. Green Chem..

[12] Aliyu OM, Awopetu JA (2007). Chromosome studies in cashew (*Anacardium occidentale* L.). Afr. J. Biotechnol..

[13] Aliyu OM (2014). Analysis of absolute nuclear DNA content reveals a small genome and intra-specific variation in Cashew (*Anacardium occidentale* L.,) *Anacardiaceae*. Silvae Genet..

[14] Mneney E, Mantell S, Bennett M (2001). Use of random amplified polymorphic DNA (RAPD) markers to reveal genetic diversity within and between populations of cashew (*Anacardium occidentale* L.). J. Hortic. Sci. Biotechnol..

[15] Archak S, Gaikwad AB, Swamy KRM, Karihaloo JL (2009). Genetic analysis and historical perspective of cashew (*Anacardium occidentale* L.) introduction into India. Genome.

[16] Jena RC, Samal KC, Pal A, Das BK, Chand PK (2016). Genetic diversity among some promising Indian local selections and hybrids of cashew nut based on morphometric and molecular markers. Int. J. Fruit Sci..

[17] Croxford AE, Robson M, Wilkinson MJ (2006). Characterization and PCR multiplexing of polymorphic microsatellite loci in cashew (*Anacardium occidentale* L.) and their cross-species utilization. Mol. Ecol. Notes.

[18] Mzena GP, Kusolwa P, Rwegasira GR, Yao N (2018). Discovery of novel Single Nucleotide Polymorphic (SNP) markers for genetic mapping of cashew (*Anacardium occidentale* L). Int. J. Agric. Environ. Bio-res..

[19] Cavalcanti JJ, Wilkinson MJ (2007). The first genetic maps of cashew (*Anacardium occidentale* L.). Euphytica.

[20] Freire FCO, Cardoso JE, Dos Santos AA, Viana FMP (2002). Diseases of cashew nut plants (*Anacardium occidentale* L.) in Brazil. Crop Prot..

[21] Wonni I, Sereme D, Ouedraogo I, Kassankagno AI, Dao I, Ouedraogo L, Nacro S (2017). Diseases of cashew nut plants (*Anacardium occidentale* L.) in Burkina Faso. Adv. Plants Agric. Res..

[22] Savadi S, Prasad P, Kashyap PL, Bhardwaj SC (2018). Molecular breeding technologies and strategies for rust resistance in wheat (*Triticum aestivum*) for sustained food security. Plant Pathol..

[23] Meyers BC, Kaushik S, Nandety RS (2005). Evolving disease resistance genes. Curr. Opin. Plant Biol..

[24] Friedman AR, Baker BJ (2007). The evolution of resistance genes in multi-protein plant resistance systems. Curr. Opin. Genet. Dev..

[25] Varshney RK, Graner A, Sorrells ME (2005). Genomics-assisted breeding for crop improvement. Trends Plant Sci..

[26] Andrews S. FastQC: a quality control tool for high throughput sequence data (2010). Available online at: http://www.bioinformatics.babraham.ac.uk/projects/fastqc.

[27] Krueger F. (2015). Trim Galore: a wrapper tool around Cutadapt and FastQC to consistently apply quality and adapter trimming to FastQ files, with some extra functionality for MspI-digested RRBS-type (reduced representation bisufite-seq) libraries. https://www.bioinformatics.babraham.ac.uk/projects/trim_galore/.

[28] Chen S, Zhou Y, Chen Y, Gu J (2018). fastp: an ultra-fast all-in-one FASTQ preprocessor. Bioinformatics.

[29] Marçais G, Kingsford C (2011). A fast, lock-free approach for efficient parallel counting of occurrences of k-mers. Bioinformatics.

[30] Ranallo-Benavidez TR, Jaron KS, Schatz MC (2020). GenomeScope 2.0 and Smudgeplot for reference-free profiling of polyploid genomes. Nat. Commun..

[31] Zimin AV, Marçais G, Puiu D, Roberts M, Salzberg SL, Yorke JA (2013). The MaSuRCA genome assembler. Bioinformatics.

[32] Seppey M, Manni M, Zdobnov EM, Kollmar M (2019). BUSCO: Assessing Genome Assembly and Annotation Completeness. Gene Prediction. Methods in Molecular Biology.

[33] Holt C, Yandell M (2011). MAKER2: an annotation pipeline and genome-database management tool for second-generation genome projects. BMC Bioinform..

[34] Camacho C, Coulouris G, Avagyan V, Ma N, Papadopoulos J, Bealer K, Madden TL (2009). BLAST+: architecture and applications. BMC Bioinform..

[35] Gene Ontology Consortium (2004). The Gene Ontology (GO) database and informatics resource. Nucleic Acids Res..

[36] Moriya Y, Itoh M, Okuda S, Yoshizawa AC, Kanehisa M (2007). KAAS: an automatic genome annotation and pathway reconstruction server. Nucleic Acids Res..

[37] Mao X, Cai T, Olyarchuk JG, Wei L (2005). Automated genome annotation and pathway identification using the KEGG Orthology (KO) as a controlled vocabulary. Bioinformatics.

[38] Kanehisa M, Goto S (2000). KEGG: Kyoto Encyclopedia of Genes and Genomes. Nucleic Acids Res.

[39] Kim D, Paggi JM, Park C (2019). Graph-based genome alignment and genotyping with HISAT2 and HISAT-genotype. Nat. Biotechnol..

[40] Kent WJ (2002). BLAT-the BLAST-like alignment tool. Genome Res..

[41] Smit, A.F.A., Hubley R., Green, P., RepeatMasker at http://repeatmasker.org

[42] Nawrocki EP, Eddy SR (2013). Infernal 1.1: 100-fold faster RNA homology searches. Bioinformatics.

[43] Griffiths-Jones S, Bateman A, Marshall M, Khanna A, Eddy SR (2003). Rfam: an RNA family database. Nucleic Acids Res..

[44] Osuna-Cruz CM, Paytuvi-Gallart A, Di Donato A, Sundesha V, Andolfo G, Aiese Cigliano R, Sanseverino W, Ercolano MR (2018). PRGdb 3.0: a comprehensive platform for prediction and analysis of plant disease resistance genes. Nucleic Acids Res..

[45] Prüfer K, Stenzel U, Dannemann M, Green RE, Lachmann M, Kelso J (2008). PatMaN: rapid alignment of short sequences to large databases. Bioinformatics.

[46] Emms DM, Kelly S (2019). OrthoFinder: Phylogenetic orthology inference for comparative genomics. Genome Biol..

[47] Edgar RC (2004). MUSCLE: multiple sequence alignment with high accuracy and high throughput. Nucleic Acids Res..

[48] Tamura K, Battistuzzi FU, Billing-Ross P, Murillo O, Filipski A, Kumar S (2012). Estimating divergence times in large molecular phylogenies. Proc Natl Acad Sci USA.

[49] Emms, D.M., Kelly, S. STAG: Species Tree Inference from All Genes. bioRxiv (2018). DO -10.1101/267914

[50] Emms DM, Kelly S (2017). STRIDE: Species tree root inference from gene duplication events. Mol. Biol. Evol..

[51] Mendes FK, Vanderpool D, Fulton B (2020). CAFE 5 models variation in evolutionary rates among gene families. Bioinformatics.

[52] Bu D, Luo H, Huo P, Wang Z, Zhang S, He Z, Wu Y, Zhao L, Liu J, Guo J, Fang S, Cao W, Yi L, Zhao Y, Kong L (2021). KOBAS-i: intelligent prioritization and exploratory visualization of biological functions for gene enrichment analysis. Nucleic Acids Res..

[53] Finkers R, van Kaauwen M, Ament K, Burger-Meijer K, Egging R, Huits H, Kodde L, Kroon L, Shigyo M, Sato S, Vosman B, van Workum W, Scholten O (2021). Insights from the first genome assembly of Onion (*Allium cepa*). G3.

[54] Simão FA, Waterhouse RM, Ioannidis P, Kriventseva EV, Zdobnov EM (2015). BUSCO: assessing genome assembly and annotation completeness with single-copy orthologs. Bioinformatics.

[55] Zeng L, Tu XL, Dai H, Han FM, Lu BS, Wang MS, Nanaei HA, Tajabadipour A, Mansouri M, Li X-L, Ji L-L, Irwin DM, Zhou H, Liu M, Zheng H-K, Esmailizadeh A, Wu DD (2019). Whole genomes and transcriptomes reveal adaptation and domestication of pistachio. Genome Biol..

[56] Bally IS, Bombarely A, Chambers AH, Cohen Y, Dillon NL, Innes DJ, Islas-Osuna MA, Kuhn DN, Mueller LA, Ophir R, Rambani A, Sherman A, Yan H (2021). The ‘Tommy Atkins’ mango genome reveals candidate genes for fruit quality. BMC Plant Biol..

[57] Zhu QH, Wang MB (2012). Molecular functions of long non-coding RNAs in plants. Genes.

[58] Millar AA (2020). The function of miRNAs in plants. Plants.

[59] Ng KLS, De De Mishra SK (2007). novo SVM classifcation of precursor microRNAs from genomic pseudo hairpins using global and intrinsic folding measures. Bioinformatics.

[60] Kaur P, Gaikwad K (2017). From genomes to GENE-omes: Exome sequencing concept and applications in crop improvement. Front. Plant Sci..

[61] Meng J, Liu D, Sun C, Luan Y (2014). Prediction of plant pre-microRNAs and their microRNAs in genome-scale sequences using structure-sequence features and support vector machine. BMC Bioinform..

[62] Fu X, Zhu W, Cai L, Liao B, Peng L, Chen Y, Yang J (2019). Improved pre-miRNAs identifcation through mutual information of pre-miRNA sequences and structures. Front. Genet..

[63] Soyturk A, Sen F, Uncu AT, Celik I, De Uncu AO (2021). novo assembly and characterization of the first draft genome of quince (*Cydonia oblonga* Mill). Sci. Rep..

[64] Lisch D (2013). How important are transposons for plant evolution?. Nat. Rev. Genet..

[65] Zhang L, Hu J, Han X, Li J, Gao Y, Richards CM, Zhang C, Tian Y, Liu G, Gul H, Wang D, Tian Y, Yang C, Meng M, Yuan G, Kang G, Wu Y, Wang K, Zhang H, Wang D, Cong P (2019). A high-quality apple genome assembly reveals the association of a retrotransposon and red fruit colour. Nat. Commun..

[66] Hammond‐Kosack, K. E., & Kanyuka, K. Resistance genes (*R* genes) in plants. eLS. (2007).

[67] Liu, S. Improved Hybrid de novo Genome Assembly, Resistance Gene Prediction and Annotation of Carrot (*Daucus carota*). A thesis submitted to North Carolina State University, (2020).

[68] Jegadeesan S, Raizada A, Dhanasekar P, Suprasanna P (2021). Draft genome sequence of the pulse crop blackgram [*Vigna mungo* (L.) Hepper] reveals potential R-genes. Sci. Rep..

[69] Wannan BS (2006). Analysis of generic relationships in *Anacardiaceae*. Blumea-Biodivers. Evolut. Biogeogr. Plants.

[70] Soltis PS, Soltis DE (2021). Plant genomes: Markers of evolutionary history and drivers of evolutionary change. Plant People Planet.

[71] Zhang Y, Zheng L, Zheng Y, Zhou C, Huang P, Xiao X, Zhao Y, Hao X, Hu Z, Chen Q, Li H, Wang X, Fukushima K, Wang G, Li C (2019). Assembly and annotation of a draft genome of the medicinal plant Polygonum cuspidatum. Front. Plant Sci..

