# Supplementary figures and images for: De novo assembly and characterization of the draft genome of the cashew (Anacardium occidentale L.)
Source: Sci Rep. 2022 Oct 28;12:18187. doi: 10.1038/s41598-022-22600-7 (PMC9616956; doi:10.1038/s41598-022-22600-7)

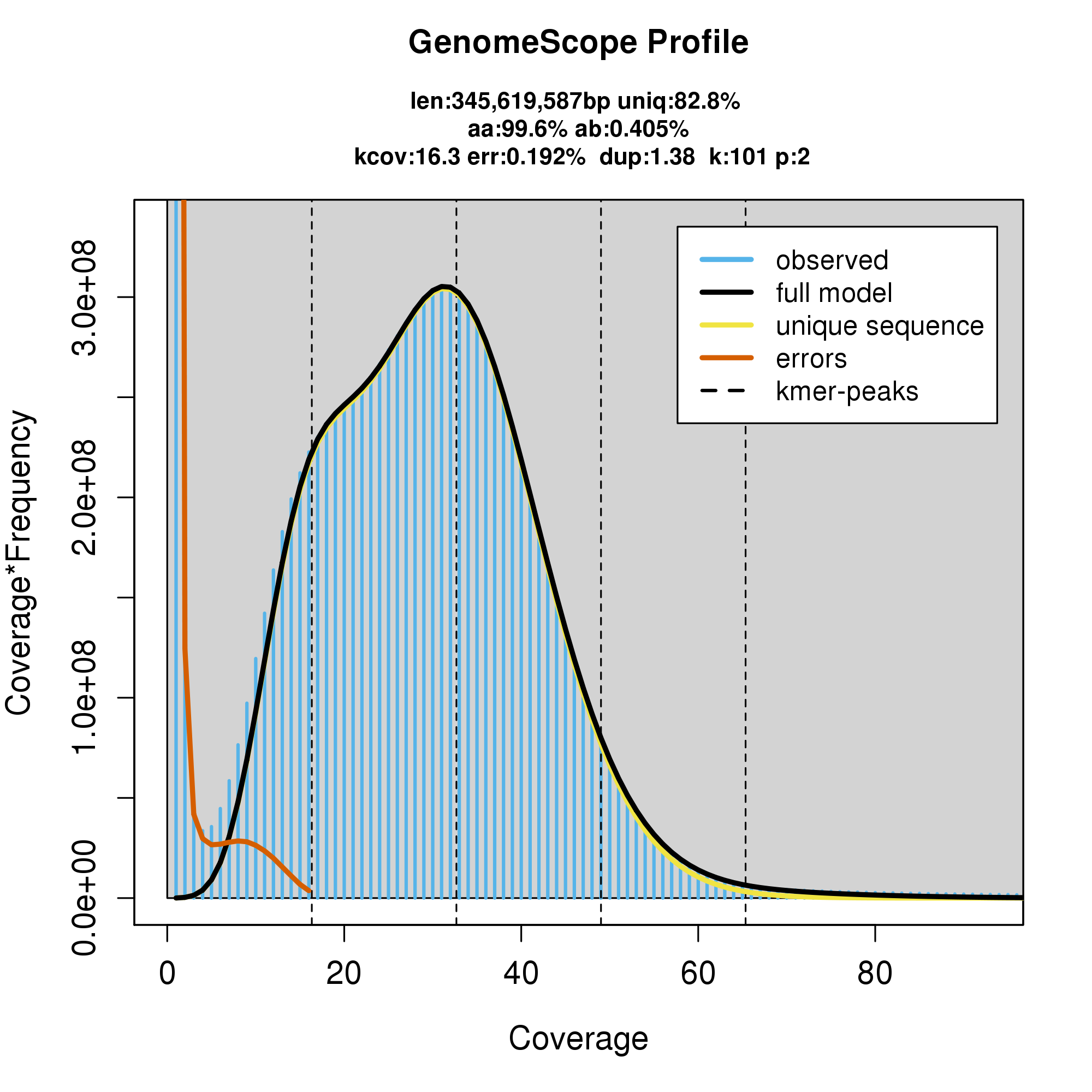

Supplement: Supplementary file 8 — Supplementary Figure S1. [file 41598_2022_22600_MOESM8_ESM.png]

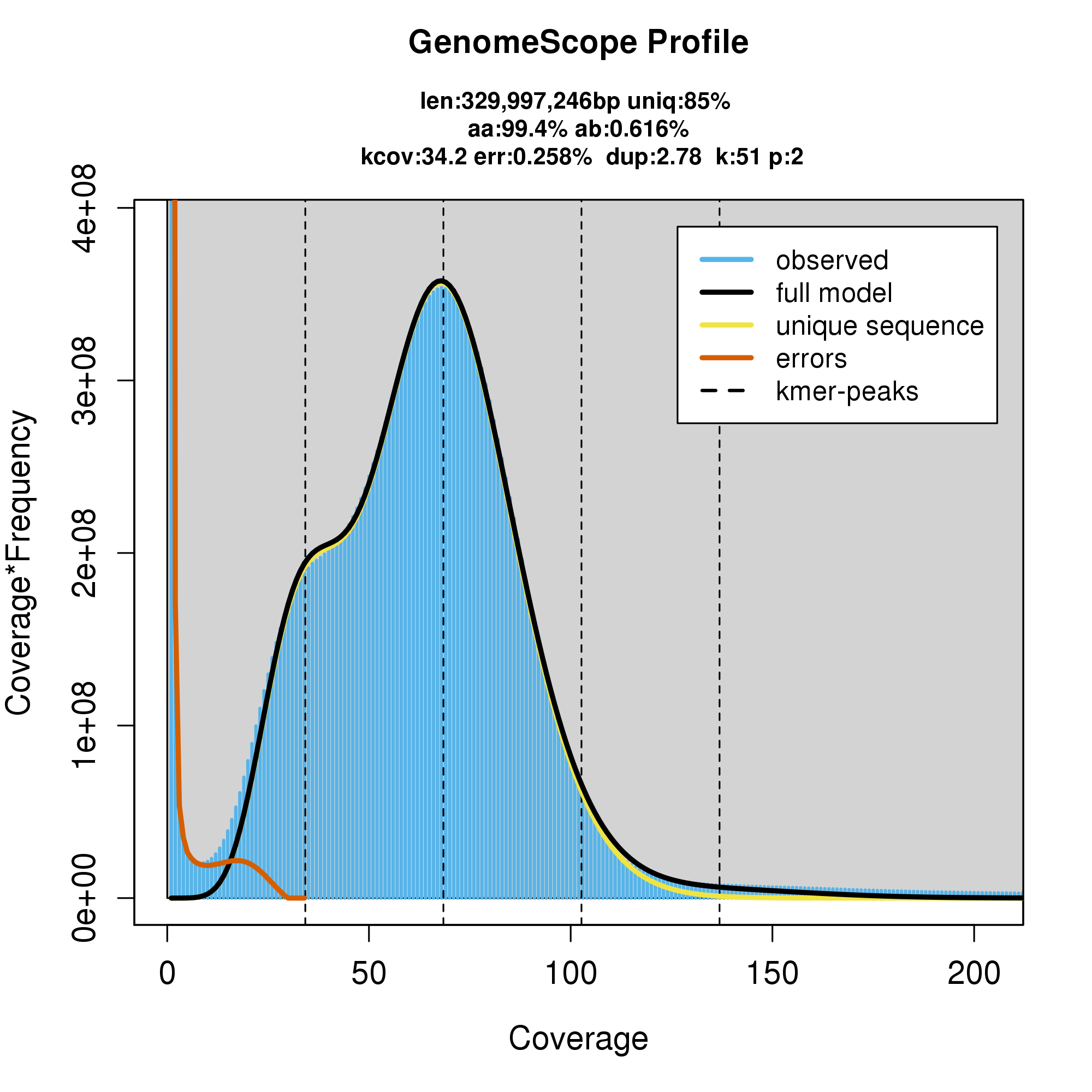

Supplement: Supplementary file 9 — Supplementary Figure S1. [file 41598_2022_22600_MOESM9_ESM.png]

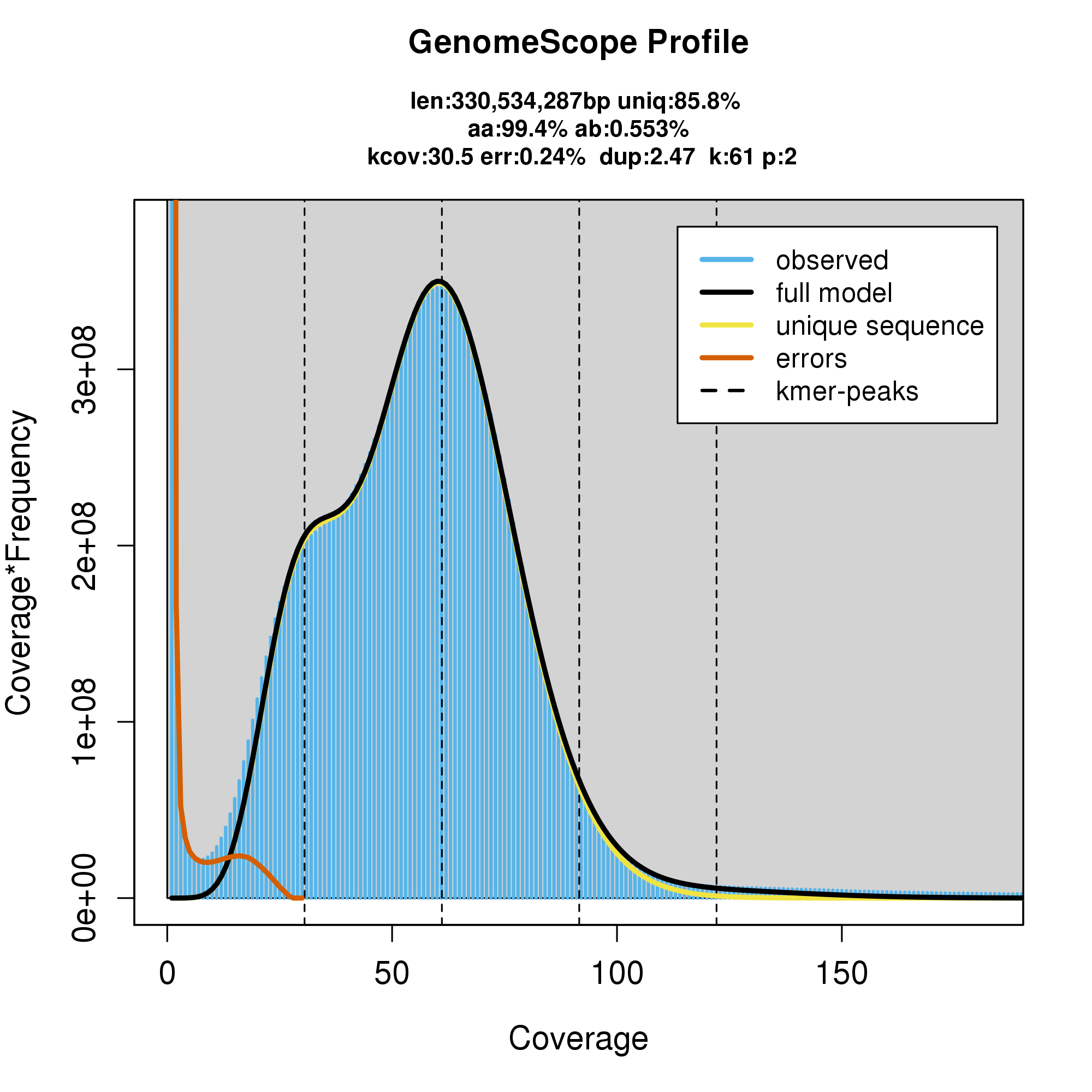

Supplement: Supplementary file 10 — Supplementary Figure S1. [file 41598_2022_22600_MOESM10_ESM.png]

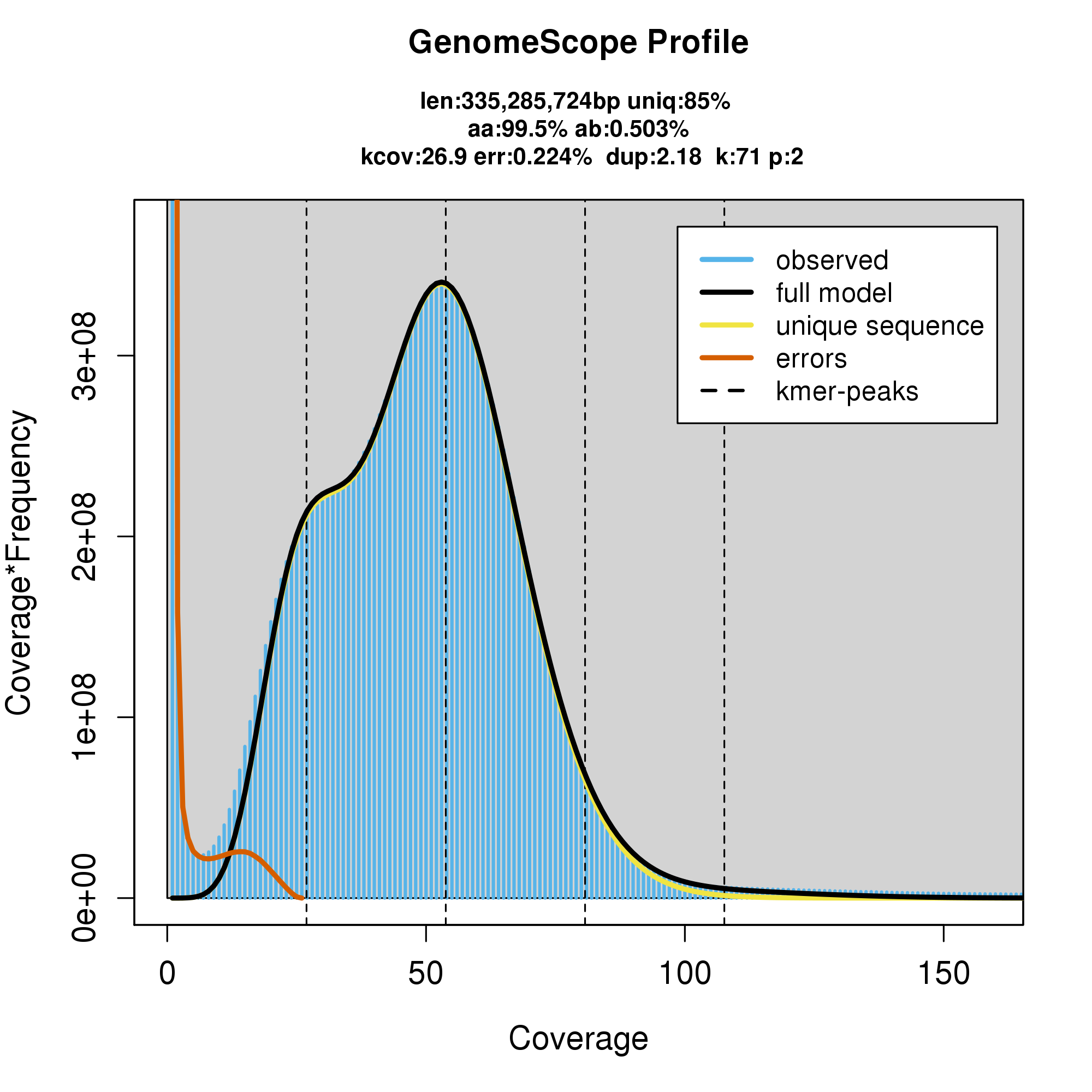

Supplement: Supplementary file 11 — Supplementary Figure S1. [file 41598_2022_22600_MOESM11_ESM.png]

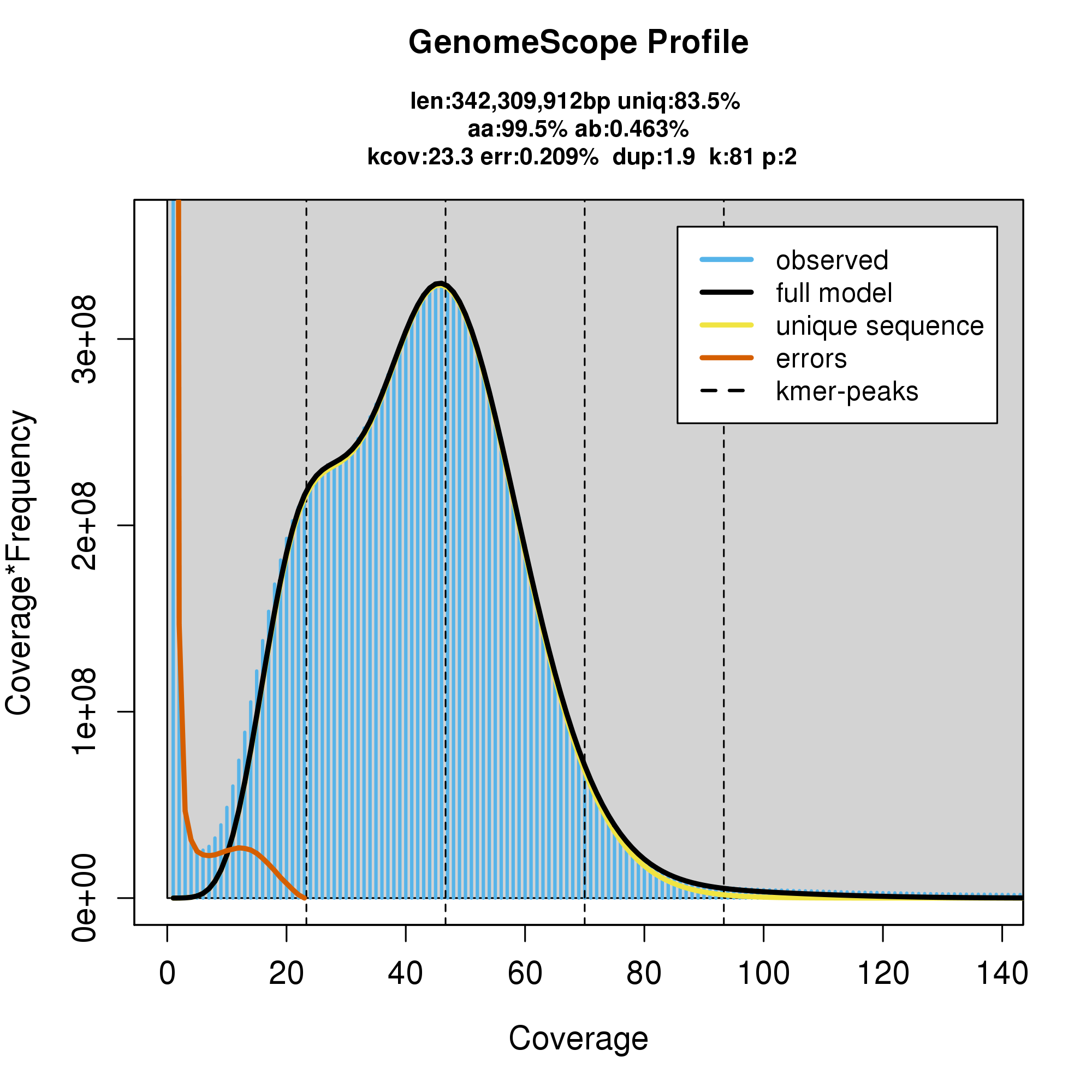

Supplement: Supplementary file 12 — Supplementary Figure S1. [file 41598_2022_22600_MOESM12_ESM.png]

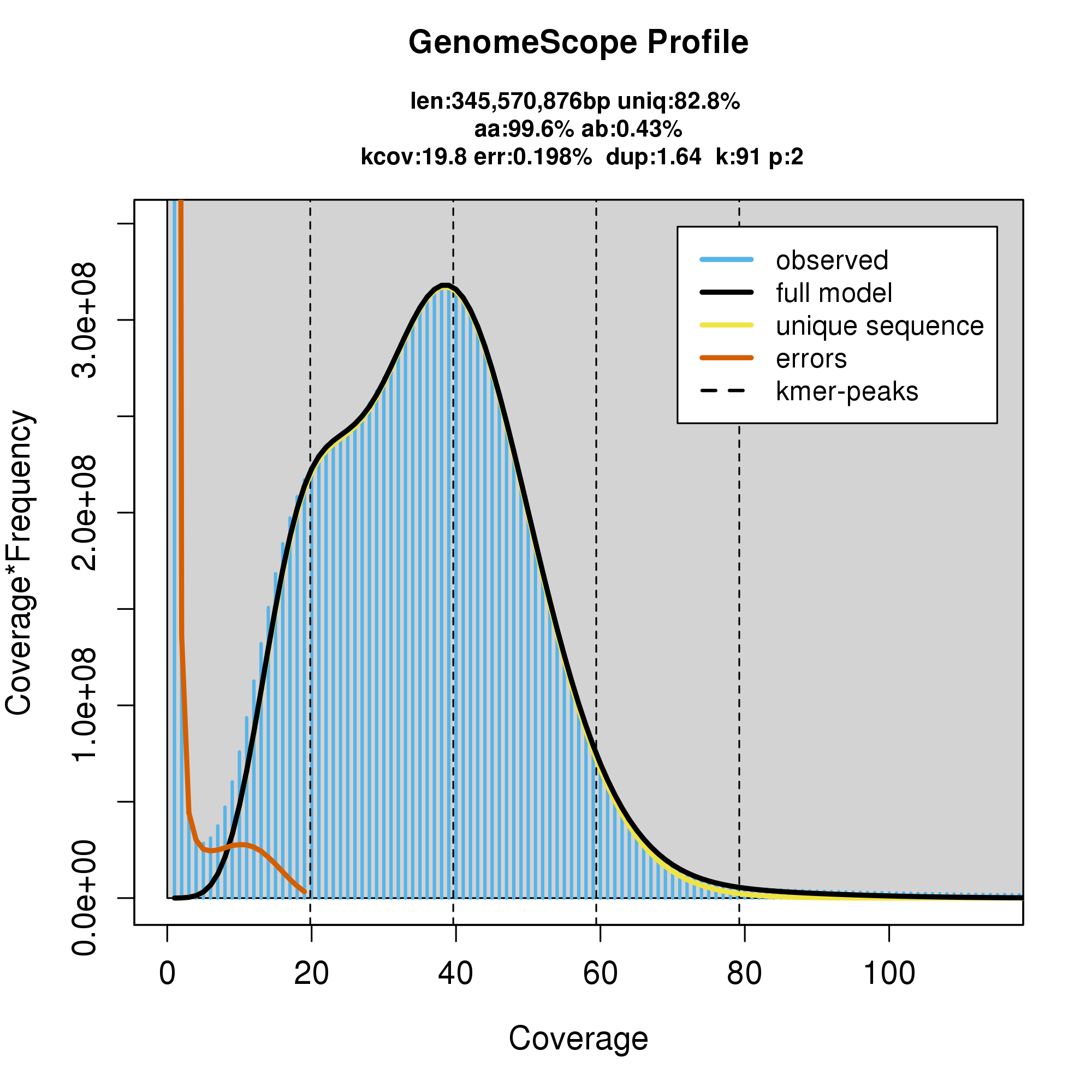

Supplement: Supplementary file 13 — Supplementary Figure S1. [file 41598_2022_22600_MOESM13_ESM.png]
